# A Prospective Study of the Association of Metacognitive Beliefs and Processes with Persistent Emotional Distress After Diagnosis of Cancer

**DOI:** 10.1007/s10608-014-9640-x

**Published:** 2014-09-11

**Authors:** Sharon A. Cook, Peter Salmon, Graham Dunn, Chris Holcombe, Philip Cornford, Peter Fisher

**Affiliations:** 1 Psychological Sciences, University of Liverpool, The Whelan Building, Brownlow Hill, Liverpool, L69 3GB UK; 2 Royal Liverpool and Broadgreen University Hospitals NHS Trust, Liverpool, UK; 3 Centre for Biostatistics, The University of Manchester, Manchester, UK

**Keywords:** Metacognitive beliefs, Breast cancer, Prostate cancer, Emotional distress, Causal predictors

## Abstract

Two hundred and six patients, diagnosed with primary breast or prostate cancer completed self-report questionnaires on two occasions: before treatment (T1) and 12 months later (T2). The questionnaires included: the Hospital Anxiety and Depression Scale; Impact of Events Scale; the Metacognitions Questionnaire-30 (MCQ-30) and the Illness Perceptions Questionnaire-revised. A series of regression analyses indicated that metacognitive beliefs at T1 predicted between 14 and 19 % of the variance in symptoms of anxiety, depression and trauma at T2 after controlling for age and gender. For all three outcomes, the MCQ-30 subscale ‘negative beliefs about worry’ made the largest individual contribution with ‘cognitive confidence’ also contributing in each case. For anxiety, a third metacognitive variable, ‘positive beliefs about worry’ also predicted variance in T2 symptoms. In addition, hierarchical analyses indicated that metacognitive beliefs explained a small but significant amount of variance in T2 anxiety (2 %) and T2 depression (4 %) over and above that explained by demographic variables, T1 symptoms and T1 illness perceptions. The findings suggest that modifying metacognitive beliefs and processes has the potential to alleviate distress associated with cancer.

## Introduction

Survival rates in cancer continue to improve. It is estimated that there are over 10 million cancer survivors in the USA (Institute of Medicine 2007), and around 2 million in the UK (Maddams et al. 2009). However, despite improved survival, cancer remains a life-threatening diagnosis which often has a profound emotional impact years after treatment has ended (Helgeson et al. 2004; Meyerowitz et al. 2008). Although emotional distress is considered a normal response around the time of cancer diagnosis, it is also common across the cancer trajectory with over a third of patients in treatment or long-term follow-up reporting clinically significant levels of distress, including anxiety and depression, that warrants intervention (Carlson et al. 2004), while life-time prevalence of cancer-related PTSD is 10–12 % for breast cancer and 20 % for other cancers (Andrykowski and Kangas 2010). In recognition of this continuing impact, health policies recommend that all patients should undergo psychological assessment at key point from diagnosis, and have prompt access to psychological support (Holland 1999; Institute of Medicine 2007; National Institute for Health and Clinical Excellence 2004).

A common and particularly influential approach to providing such support in cancer is cognitive behaviour therapy (CBT) (Watson et al. 2007; Williams and Dale 2006). It is based on the premise that negative illness appraisal (i.e. negative thoughts about cancer and its consequences) instigates and maintains distress. Research has indeed begun to show that negative thoughts about cancer are associated with current (Cook et al. 2014; Whitaker et al. 2008) or later distress (Llewellyn et al. 2007; Millar et al. 2005). However, negative thoughts are common, and especially so in people newly diagnosed with cancer, yet not everyone becomes distressed as a result of these thoughts. Furthermore, the focus of CBT on challenging negative thoughts is hard to reconcile with the clinical reality of an often uncertain future, and recent research has indicated that many patients, especially in the early stages of cancer treatment, find engaging with the negative content of their thoughts about cancer too difficult or distressing (Baker et al. 2012). Meta-analytic studies of CBT and other psychotherapeutic interventions in cancer have produced mixed results, with one recent meta-analysis concluding that small to moderate effect sizes are typical (Faller et al. 2013). Collectively these studies indicate that there is considerable room for improvement in psychotherapeutic effectiveness.

Focusing on understanding the mechanisms underlying the maintenance of emotional distress after cancer diagnosis may help to enhance the efficacy achieved by psychological interventions (Faller et al. 2013). Possible mechanisms of change are suggested by the metacognitive model of emotional disorder (Wells and Mathews 1994, 1996). This model asserts that it is not the negative content of thoughts about cancer that explains why distress is maintained but how the individual responds to those thoughts. For most people, periods of distress in relation to cancer, or any other stressor, are transitory. However, the metacognitive model proposes that people are vulnerable to persistent distress when they hold maladaptive metacognitive beliefs which guide them towards a particularly toxic style of sustained and inflexible conscious processing of negative thoughts and feelings about their cancer. This is called the cognitive attentional syndrome (CAS), and includes cognitive processes such as persistent worry and rumination, focussing of attention on threat, and maladaptive coping strategies (e.g. avoidance or thought suppression). Such processes ultimately ‘backfire’ by sustaining negative thinking and the sense of threat rather than allowing such experiences to fade naturally. According to this model, negative content of thoughts about cancer may trigger metacognitive beliefs and activate the CAS or may be a product of worry and rumination, but have no direct causal role in maintaining emotional distress. Modifying metacognitive beliefs and interrupting the CAS has been effective in treating depression and a range of anxiety disorders (see Wells 2009 for a review). In addition, metacognitive beliefs have been associated with heightened emotional distress in physical health populations including: Parkinson’s disease (Allott et al. 2005), chronic fatigue (Maher-Edwards et al. 2011), breast cancer (Cook et al. 2014; Thewes et al. 2013) and prostate cancer (Cook et al. 2014).

Our previous research (Cook et al. 2014), indicated that metacognitive beliefs (specifically ‘positive beliefs about worry’ and ‘negative beliefs about worry’) were associated with concurrent symptoms of anxiety, depression and trauma among patients recently diagnosed with breast or prostate cancer, and that they explained additional variance in these outcomes after controlling for age, gender and negative content of thoughts about cancer (i.e. negative illness perceptions). Structural equation modelling found evidence consistent with the central predictions of the metacognitive model that these beliefs cause and maintain distress directly, but also indirectly by driving worry. These findings provide the first evidence consistent with the theory that metacognitive beliefs underlie emotional distress experienced by cancer patients. However in order to provide more compelling evidence of a causal role for metacognitive beliefs in maintaining emotional distress after cancer, prospective research is needed to demonstrate a temporal relationship. Consequently, the aim of this study is to explore whether metacognitive beliefs measured shortly after diagnosis (T1) predict symptoms of anxiety, depression and trauma 12 months later (T2) and to explore whether they add to the variance explained over and above previously implicated variables including T1 symptoms and content of thoughts about cancer (i.e. T1 illness perceptions). Specifically we hypothesised that:

1. Metacognitive beliefs assessed around the time of diagnosis will prospectively predict variance in anxiety depression and trauma 12 months later
2. Metacognitive beliefs assessed around the time of diagnosis will add to the variance explained in T2 anxiety, depression and trauma symptoms over and above demographic variables, T1 symptoms and T1 illness perceptions

## Methods

### Design

A prospective cohort design included a pre-treatment baseline and 12 month follow-up. The study was approved by the NHS North West 5 Research Ethics Committee (reference: 09/H1010/70).

### Procedure

From February 2010 to May 2011, 229 patients were consecutively recruited through two pre-treatment cancer clinics at a National Health Service (NHS) teaching hospital in North-West England. Inclusion criteria were a diagnosis of primary breast or prostate cancer and at least 18 years of age. Patients with recurrent or metastatic disease and those judged by the clinical team or researcher to be too distressed or confused to give informed consent were excluded. T1 data was obtained from self-report questionnaires and medical records shortly after diagnosis before primary treatment. Follow-up data (T2) was collected 12 months later through self-report questionnaires mailed to participants’ homes.

## Measures

All Measures were Assessed Both at T1 and T2

The Hospital Anxiety and Depression Scale [HADS, (Zigmond and Snaith 1983)] assessed the primary outcomes of anxiety and depression. The HADS is the most widely used measure of anxiety and depression in physical illness and has been extensively validated for cancer populations (Luckett et al. 2010; Norton et al. 2013; Vodermaier and Millman 2011). It consists of fourteen items (seven in each subscale) scored on a 4-point scale. The subscale scores range from 0 to 21, high scores indicating greater anxiety or depression and scores of 8 or more indicating clinically significant levels. In the current sample, both subscales had good internal consistency (Cronbach’s α:.84/.88 for T1/T2 depression; .88/.89 for T1/T2 anxiety).

Trauma symptoms were assessed using the Impact of Events Scale [IES; (Horowitz et al. 1979)]. This 15-item measure yields a total score of 0–75, with high scores indicating greater trauma. It has been validated as a screening measure of stress reactions after a range of traumatic events (Sundin and Horowitz 2002). There is no consensus on a cut-off score for clinically significant trauma. However, a score of 27 or more provided an overall correct classification rate of 80 % in a large sample of motor vehicle accident survivors comprising both genders (Coffey et al. 2006), and has previously been used in cancer (Purnell et al. 2011). Internal consistency of the IES was excellent at both time points in the current sample (Cronbach’s α: .90/.94).

The revised Illness Perceptions Questionnaire [IPQ-R (Moss-Morris et al. 2002)] was used to assess participants’ appraisal of their cancer. It assessed thoughts, ideas and beliefs about cancer in several distinct areas, including: ‘identity’ (the number of symptoms participants attribute to their cancer); ‘chronic timeline’ (how long they think it will last); ‘cyclical timeline’ (the perception that symptoms are cyclical); ‘consequences’ (the perception that cancer has negative consequences on life); ‘personal control’ and ‘treatment control’ (the beliefs that it can be controlled or cured by their own actions or by treatment, respectively); ‘illness coherence’ (the extent to which they feel they understand their illness); *‘*emotional representation’ (their emotional response to their illness); and beliefs about causality. As the IPQ-R was included to assess the relative importance of patients’ illness appraisal, the emotional representation subscale was disregarded. The final section of the IPQ-R assesses patients’ causal attributions. These items are typically not summed as a single scale but may be analysed as separate items or as groups devised on the basis of theory (Moss-Morris et al. 2002). As only psychological and/or behavioural attributions have contributed to variance in quality of life (Scharloo et al. 2010) or emotional distress (Kulik and Kronfeld 2005; Traeger et al. 2009) after diagnosis of cancer, the seven causal items which reflect these attributions (i.e. ‘my own behaviour’, ‘my mental attitude’, ‘stress or worry’, ‘my emotional state’, and ‘my personality’ ‘family problems or worries’ and ‘overwork’) were used to generate a single causal subscale (‘psychological attributions’) and the remaining causal items were disregarded. With the exception of the identity subscale (in which the items are dichotomous), all IPQ-R items are scored 1–5 with higher subscale scores indicating greater endorsement of that illness perception. In the current sample, Cronbach’s α: of the IPQ-R subscales ranged from .64 (‘personal control’) to .82 (‘chronic timeline’/’cyclical timeline’) at time 1 and from .63 (‘treatment control’) to .89 (‘cyclical timeline’) at time 2, indicating relatively poor to good internal consistency.

Metacognitive beliefs were measured using the Metacognitions Questionnaire 30- [MCQ-30 (Wells and Cartwright-Hatton 2004)]. The MCQ-30 was developed specifically to assess key components of the metacognitive model of emotional disorder. It comprises five subscales: *‘*positive beliefs about worry’; ‘negative beliefs about worry’; ‘cognitive confidence’; ‘need to control thoughts’; and ‘cognitive self-consciousness’. For each, items are scored 1–4, yielding a total score of 6–24. High scores indicate more positive or negative beliefs about worry, reduced confidence in memory, greater belief in the need to control thoughts and an increased tendency towards self-focussed attention, respectively. In the current sample, Cronbach’s α: of MCQ-30 subscales ranged from .73 (‘need to control thoughts’) to .89 (*‘*positive beliefs about worry’) at T1 and from .79 (‘need to control thoughts’) to .91 (*‘*positive beliefs about worry’) at T2, indicating adequate to excellent internal consistencey.

### Analysis

The data were analysed using SPSS Version 20. As <2 % of the data were missing at the scale level, and these were confirmed to be missing completely at random, missing scores were imputed using the SPSS Expectation–Maximisation algorithm (Little and Rubin 1987).

As not all scales were normally distributed, nonparametric statistics (Mann–Whitney or Kruskal–Wallis) were used to compare participants who completed both assessments with those who only completed T1 assessment on age group (divided above and below the median age), gender, educational level, perceived emotional social support, stage of disease, T1 HADS and IES scores. They were also used to examine the relationship of each T2 outcome with demographic, clinical and social support variables. Where significant associations with T2 outcomes were found (*p* < .05), the relevant variables were entered as demographic control variables in the first step of subsequent regression analyses.

Initially, the IPQ-R and MCQ-30 were analysed in parallel to identify which subscales within each measure independently predicted each T2 outcome. For the IPQ-R, hierarchical multiple regression analyses were first used to identify the T1 subscales associated with each T2 outcome (anxiety, depression and trauma) after controlling for demographic variables (Analysis 1 for each outcome). These analyses were then repeated, using just the significant IPQ-R subscales from Analysis 1, and also controlling for T1 symptoms of anxiety, depression or trauma (Analysis 2 for each outcome). As we had no a priori theory about which subscales would independently predict T2 outcomes, the IPQ-R subscales were included in each analysis using stepwise rather than forced entry. The subscales identified as independent predictors in Analysis 2 for each outcome were then entered as variables in Analysis 3 for that outcome (see below).

This sequence of analyses was repeated for the MCQ-30, thereby testing hypothesis 1. We first identified the T1 MCQ-30 subscales that independently predict T2 outcomes after controlling for demographic variables (Analysis 1 for each outcome), and then entered these in a further analysis also controlling for T1 symptoms of anxiety, depression or trauma (Analysis 2 for each outcome). As with the IPQ-R analyses, as we had no a priori theory about which subscales would independently predict T2 outcomes, MCQ-30 subscales were included in each analysis using stepwise rather than forced entry. The subscales identified as independent predictors in Analysis 2 for each outcome were then entered as variables in Analysis 3 for that outcome (see below), which tested hypothesis 2.

Final hierarchical multiple regression analyses (Analysis 3 for each outcome) assessed whether the T1 MCQ-30 subscales which had been identified as significant predictors in Analysis 2 (see above) were able to predict variance in T2 outcomes over and above that explained by demographic variables, T1 symptoms and the negative content of thoughts about cancer at Time 1 (i.e. IPQ-R subscales identified as significant predictors in Analysis 2). This final analysis used forced entry and bootstrapped sampling to ensure findings were robust.

## Results

### Completers Versus Non Completers

Of the 229 participants who completed T1 questionnaires 206 (90 %) also completed the assessment 12 months later. No significant differences between completers and non-completers were apparent on T1 HADS and IES scores, age, gender, education, or tumour grade. However non-completers were more likely than completers to report low levels of perceived emotional support at T1 (52 vs. 31 % *p* = .034).

### Sample Characteristics

The baseline demographic and clinical characteristics of the final sample (N = 206) are shown in Table 1. Women with breast cancer and younger patients were more anxious at T2 (U = −3,269.5, *p* < .001, r = −.27; U = −3,721, *p* < .001. r = .26), and reported more trauma symptoms (U = 3,636, *p* = .003, r = −.21; U = 3,638, *p* < .001, r = .27) than did men with prostate cancer or older patients. Women with breast cancer also reported more symptoms of depression at T2 than did men with prostate cancer (U = 3,857.5, *p* = .014, r = .17). No outcome was related to education, perceived emotional support or tumour grade. Therefore just age and gender were used as demographic covariates in subsequent analyses. The levels of anxiety, depression and trauma symptoms at both time points are shown in Table 2. Both anxiety and trauma symptoms significantly declined over time, whereas depressive symptoms significantly increased.

**Table 1:** Sample characteristics at time 1 (N = 206)

Age	
Mean (SD)	61.5 (9.0)
Range	39–85
*Gender*	
Female	133
Male	73
*Marital status*	
Married/co-habiting	139
Single/divorced/widowed	62
Live alone	37
*Education*	
None	76
School qualifications or higher	121
*Employment*	
Employed (full/part-time)	79
Retired	92
Retired (health)	14
Homemaker	9
Unemployed	9
*Cancer diagnosis*	
Breast	133
Prostate	73
*Tumour grade*	
Low	54
Intermediate	97
High	52

N.B. Missing data: marital status n = 5; live alone n = 3; education n = 9; employment n = 3; tumour grade n = 3

**Table 2:** Distribution of anxiety, depression and trauma scores at both time-points

	Time 1		Time 2		T1–T2 difference
	Mean (SD)	Median (IQR)	Mean (SD)	Median (IQR)	
Anxiety	7.6 (4.4)	7.5 (4–11)	6.2 (4.5)	5 (3–9)	Z = −4.6; r = −.23; *p* = .000
Depression	3.3 (3.3)	2 (1–5)	4.1 (3.9)	3 (1–6.6)	Z = 3.1; r = .015; *p* = .002
Trauma	29.4 (16.9)	31 (14–42.3)	21.2 (18.9)	17 (3.3–35)	Z = −6.5; r = −.32; *p* = .000

### Association of T1 Illness Perceptions with T2 Anxiety, Depression and Trauma

Regression of emotional distress on the IPQ-R subscales (Table 3) indicated that illness perceptions predicted between 10 % (trauma) and 12 % (anxiety) of the variance in T2 outcomes after controlling for age and gender (Analysis 1) and between 2 % (trauma) and 3 % (anxiety and depression) after also controlling for T1 symptoms (Analysis 2). The final models from Analysis 2 indicated that, perceived lack of personal control and negative perception of the consequences of cancer predicted T2 anxiety (1 and 2 % respectively), while poor understanding of the illness (‘illness coherence’) predicted T2 depression and trauma. These IPQ- R subscales were therefore used to control for content of thoughts about cancer in the final hierarchical multiple regression analyses.

**Table 3:** Final models of the variance in T2 anxiety, depression and trauma predicted by T1 illness perceptions after controlling for age and gender (Analysis 1) and age, gender and T1 levels of symptoms (Analysis 2)

	T2 anxiety				T2 depression				T2 trauma			
	R^2^ change	Beta	T	*p*	R^2^ change	Beta	T	*p*	R^2^ change	Beta	T	*p*
*Analysis 1*												
Constant			3.05	.003			3.13	.002			4.74	.000
*Step 1–demographics*	13 %^***^				5 %^**^				14 %^***^			
Gender		−.19	−3.04	.003		−.13	−1.94	.054		−.14	−2.28	.023
Age		−.21	−3.28	.001		−.11	−1.62	.106		−.27	−4.19	.000
*Step 2–IPQ-R*	12 %^***^				11 %^**^				10 %^***^			
Identity		ns	ns	ns		ns	ns	ns		ns	ns	ns
Cyclical timeline		ns	ns	ns		ns	ns	ns		ns	ns	ns
Chronic timeline		ns	ns	ns		ns	ns	ns		ns	ns	ns
Consequences		.22	3.41	.001		.19	2,75	.006		.19	2.92	.004
Illness coherence		ns	ns	ns		−.18	−2.73	.007		−.24	−3.76	.000
Psychological attributions		.19	2.97	.003		ns	ns	ns		ns	ns	ns
Personal control		−.14	−2.18	.030		−.13	−2.02	.045		ns	ns	ns
Treatment control		ns	ns	ns		ns	ns	ns		ns	ns	ns
R^2^	25 %				16 %				24 %			
Adj R^2^	23 %				13 %				23 %			
*Analysis 2*												
Constant			2.79	.006			4.61	.000			4.23	.000
*STEP 1–demographics*	13 %^***^				5 %^**^				14 %^***^			
Gender		−.03	−.42	.675		−.08	−1.36	.177		−.04	−.65	.516
Age		−.17	−2.99	.003		−.15	−2.41	.017		−.22	−3.69	.000
*Step 2—T1 symptoms*	25 %^***^				21 %^***^				23 %^***^			
T1 Anxiety		.49	8.03	.000		–	–	–		–	–	–
T1 depression		–	–	–		.44	7.25	.000		–	–	–
T1 trauma		–	–	–		–	–	–		.46	7.40	.000
*Step 2–IPQ-R* ^*##*^	3 %^*^				3 %^**^				2 %^*^			
Consequences		.13	2.17	.032		ns	ns	ns		ns	ns	ns
Illness coherence		–	–	–		−.16	−2.65	.009		−.13	−2.23	.025
Psychological attributions		ns	ns	ns		–	–	–		–	–	–
Personal control		−.11	−2.08	.039		ns	ns	ns		–	–	–
R^2^	41 %				29 %				39 %			
Adj R^2^	39 %				27 %				37 %			

R^2^ change shows increment in variance explained when each set of variables was entered sequentially; beta, T and *p* are from the final model containing variables from all steps

N.B. IPQ-R subscales entered using stepwise method

*ns* non significant, data not available using stepwise methods

* *p* < .05 , ** *p* < .01, *** *p* < .001

^##^Only subscales found to be significant predictors in Analysis 1 were entered. – indicates variable not included

### Association of T1 Metacognitive Beliefs with T2 Anxiety, Depression and Trauma

The results of the hierarchical multiple regression analyses to test hypothesis 1 are shown in Table 4. After controlling for age and gender (Analysis 1), metacognitive beliefs explained an additional 19 % of the variance in T2 anxiety, 15 % of the variance in T2 depression and 14 % of the variance in T2 trauma. In all cases ‘negative beliefs about worry’ made the largest individual contribution of all the predictors with ‘cognitive confidence’ also making a significant individual contribution. For anxiety, ‘positive beliefs about worry’ was a further significant individual predictor of T2 symptoms. After controlling for T1 symptoms as well as demographic variables (Analysis 2), metacognitive beliefs continued to predict a small but significant proportion of variance in each outcome. It added a significant 2 % to the variance in T2 anxiety, 5 % to the variance in T2 depression and 1 % to the variance in T2 trauma. In each case, ‘cognitive confidence’ was the only MCQ-30 subscale that continued to make a significant individual contribution to the variance explained, and consequently this variable was the only metacognitive variable entered into the final set of analyses (Analysis 3).

**Table 4:** Final models of the variance in T2 anxiety, depression and trauma predicted by T1 metacognitive beliefs after controlling for age and gender (Analysis 1) and age, gender and T1 levels of symptoms (Analysis 2)

	T2 anxiety				T2 depression				T2 trauma			
	R^2^ change	Beta	T	*p*	R^2^ change	Beta	T	*p*	R^2^ change	Beta	T	*p*
*Analysis 1*												
Constant			2.98	.003			1.19	.236			3.64	.000
Step 1–demographics	13 %^***^				5 %^**^				14 %^***^			
Gender		−.16	−2.67	.008		−.10	−1.60	.112		−.11	−1.70	.091
Age		−20	−3.39	.001		−.10	−1.62	.107		−.26	−4.24	.000
*Step 2–MCQ-30*	19 %^**^				15 %^***^				14 %^***^			
POS		.17	2.58	.011		ns	ns	ns		ns	ns	ns
NEG		.28	3.90	.000		.25	3.64	.000		.28	4.31	.000
CC		.12	2.00	.047		.22	3.34	.001		.17	2.66	.008
NC		ns	ns	ns		ns	ns	ns		ns	ns	ns
CSC		ns	ns	ns		ns	ns	ns		ns	ns	ns
R^2^	32 %				20 %				27 %			
Adj R^2^	30 %				18 %				26 %			
*Analysis 2*												
Constant			3.70	.000			2.34	.020			3.19	.002
*Step 1–demographics*	13 %^***^				5 %^**^				14 %^***^			
Gender		−.03	−.54	.590		−.09	−1.42	.158		−.04	−.62	.537
Age		−.20	−3.62	.000		−.13	−2.18	.030		−.21	−3.51	.001
*Step 2—T1 symptoms*	25 %^***^				21 %^***^				23 %^***^			
T1 anxiety		.50	8.22	.000		–	–	–		–	–	–
T1 depression		–	–	–		.42	7.06	.000		–	–	–
T1 trauma		–	–	–		–	–	–		.47	7.44	.000
*Step 3—MCQ-30* ^*##*^	2 %^*^				5 %^***^				1 %^*^			
POS		ns	ns	ns		–	–	–		–	–	–
NEG		ns	ns	ns		ns	ns	ns	ns	ns	ns	ns
CC		.14	2.53	.012		.23	3.85	.000		.13	2.16	.032
R^2^	40 %				31 %				38 %			
Adj R^2^	39 %				30 %				36 %			

R^2^ change shows increment in variance explained when each set of variables was entered sequentially; beta, T and* p *are from the final model containing variables from all steps

N.B. MCQ-30 subscales entered using stepwise method. MCQ-30 subscales: positive beliefs about worry (POS); negative beliefs about the danger and uncontrollability of worry (NEG); cognitive confidence (CC); need for control (NC); cognitive self-consciousness (CSC)

*ns* non significant, data not available using stepwise methods

* *p* < .05, ** *p* < .01, *** *p* < .001

^##^Only subscales found to be significant predictors in Analysis 1 were entered. – indicates variable not included

### Predictive ability of T1 Metacognitive Beliefs Over and Above: Demographic Variables, T1 Symptoms and Content of Thoughts about Cancer

The results of the hierarchical multiple regression analyses to test the second hypothesis (Analysis 3) are shown in Table 5. For anxiety and depression, ‘cognitive confidence’ added a significant 2 and 4 % respectively to the variance in T2 symptoms over and above demographic variables, T1 symptoms and content of thoughts about cancer (i.e. relevant T1 illness perceptions). For anxiety, younger age, baseline symptoms, perceived lack of personal control and low cognitive confidence each made a significant individual contribution to the final model, which accounted for 42 % of the variance in T2 symptoms. For depression, just younger age, baseline symptoms and low cognitive confidence made significant independent contributions to the final model, which accounted for 33 % of the variance in T2 symptoms.

**Table 5:** Final models of the variance in T2 anxiety, depression and trauma predicted by T1 metacognitive beliefs after controlling for age, gender, T1 level of symptoms and T1 illness perceptions (Analysis 3)

	T2 anxiety				T2 depression				T2 trauma			
	R^2^ change	Beta	T	*p*	R^2^ change	Beta	T	*p*	R^2^ change	Beta	T	*p*
*Analysis 3*												
Constant			2.50	.013			2.98	.003			3.66	.000
*Step 1–demographics*	13 %^***^				5 %^**^				14 %^***^			
Gender		−.04	−.63	.527		−.09	−1.50	.136		−.05	−.79	.430
Age		−.18	−3.06	.003		−.14	−2.29	.023		−.22	−3.73	
*Step 2—T1 symptoms*	25 %^***^				21 %^***^				23 %^***^			
T1 anxiety		.46	7.49	.000		–	–	–		–	–	–
T1 Depression		–	–	–		.41	6.86	.000		–	–	–
T1 trauma		–	–	–		–	–	–		.44	6.77	.000
*Step 3—IPQ-R* ^*##*^	3 %^**^				3 %^**^				2 %^*^			
Consequences		.12	1.94	.054		–	–	–		–	–	–
Illness coherence		–	–	–		−.11	−1.83	.068		−.11	−1.90	.058
Personal control		−.11	−2.09	.038		–	–	–		–	–	–
*Step 4—MCQ-30* ^##^	2 %^*^				4 %^**^				1 %^ns^			
CC		.13	2.32	.02		.20	3.31	.001		.11	1.78	.076
*Model summary*												
R^2^	42 %				33 %				39 %			
Adj R^2^	41 %				31 %				37 %			

R^2^ change shows increment in variance explained by each step; beta, T and* p* are from the final model containing variables from all steps

N.B. All variables entered using forced entry method. MCQ-30 subscales: cognitive confidence (CC)

^##^Only subscales found to be significant predictors in Analysis 2 were entered. – indicates variable not included

* *p* < .05, ** *p* < .01, *** *p* < .001

In the case of trauma, ‘cognitive confidence’ did not make any significant contribution to the variance explained in T2 symptoms after controlling for demographic variables, T1 symptoms and T1 illness perceptions (‘illness coherence’). In fact, younger age and T1 symptoms were the only variables to make a significant individual contribution to the final model, which accounted for 39 % of the variance.

## Discussion

This is the first study to explore whether metacognitive beliefs soon after cancer diagnosis, and before active treatment (T1), predict emotional distress 12 months later (T2). T1 metacognitive beliefs predicted T2 anxiety, depression, and trauma after controlling for age, gender and T1 symptoms, thus supporting hypothesis 1. This finding builds on previous research in non-clinical populations in which metacognitive beliefs prospectively predicted levels of anxiety and depression two (Weber and Exner 2013), three (Papageorgiou and Wells 2009; Hjemdal et al. 2013) and 6 months (Yilmaz et al. 2011) later, after controlling for age, gender and T1 levels of symptoms.

Before controlling for T1 symptoms, metacognitive beliefs explained a greater proportion of variance in T2 anxiety, depression and trauma than did illness perceptions. Furthermore the illness perception subscales that were predictive (‘consequences’, ‘personal control’, ‘psychological attributions’, ‘illness coherence’) could be considered to be markers for worry or rumination in that they may be the outcome of these processes. Of the five MCQ-30 subscales included in Analysis 1, two (‘negative beliefs about worry’ and ‘cognitive confidence’) independently predicted T2 anxiety, depression and trauma, with a third (‘positive beliefs about worry’) also significantly contributing to the variance in anxiety. In all three cases ‘negative beliefs about worry’ made the largest individual contribution, as would be predicted by the metacognitive model of emotional disorder (Wells 2009). These findings are also consistent with those of (Yilmaz et al. 2011) who reported that, in their non-clinical sample, ‘negative beliefs about worry’ predicted levels of anxiety and depression 6 months later. However, in the current study when T1 levels of distress were controlled, the relationship of ‘negative beliefs about worry’ with anxiety and depression was no longer significant. Instead ‘cognitive confidence’ was the only metacognitive variable to contribute to variance. The reasons for this are not clear.

One possibility is that this finding is due to the limitations of using hierarchical regression in a prospective study design where baseline emotional distress inevitably predominates in predicting future distress. Previous research (Cook et al. 2014) has demonstrated a strong cross-sectional association of T1 symptoms of anxiety, depression and trauma with metacognitive beliefs and processes. Consequently, as the metacognitive beliefs and processes that we measured to predict T2 distress also (according to theory) cause T1 distress, there is likely to be considerable overlap in the variance in T2 distress explained by T1 symptoms and metacognitive beliefs, leading to underestimation of the importance of the putative causal variables. That is, by controlling for baseline symptoms we may be masking the effect of the beliefs and processes that underlie its maintenance. To resolve this dilemma, approaches to analysis are required that can distinguish putatively causal effects arising from metacognitive beliefs and processes (causing symptoms of distress to be maintained) from the confounding effect resulting from symptom maintenance. Such differentiation is not feasible using standard hierarchical regression but may be possible using structural equation modelling techniques to model the effect of change in metacognitive beliefs on change in emotional distress.

As well as being able to explain more of the variance in T2 distress than did illness perceptions in Analysis 1, metacognitive beliefs (‘cognitive confidence’) were also able to explain additional variance in anxiety and depression over and above age, gender, T1 symptoms, and T1 illness perceptions (Analysis 3). This supports hypothesis 2 for these two outcomes. However, for trauma, metacognitive beliefs (‘cognitive confidence’) no longer significantly predicted T2 symptoms after including T1 illness perceptions (‘illness coherence’) in the analysis (Analysis 3). However, it should be noted that the proportion of variance in T2 trauma explained by ‘cognitive confidence’ is unchanged between Analysis 2 (controlling for T1 trauma) and Analysis 3 (controlling for T1 trauma and T1 ‘illness coherence’). Furthermore, there is little difference in the variance explained by ‘cognitive confidence’ in trauma (1 %) and in anxiety (2 %). Therefore this apparent discrepancy may be an artefact of the present data. To our knowledge this is the first study to explore the prospective relationship between metacognitive beliefs (as measured by the MCQ-30) and trauma symptoms, making it difficult to judge the reliability of this finding.

One limitation of the study is the restriction of the sample to breast and prostate cancer patients. These populations were selected because they represent the largest tumour groups in each gender and have a broadly similar prognosis. However this means it is not possible, in this sample, to separate out any effects that may be due to tumour group or gender. Furthermore, we cannot assume that the predictive effects found in this study would generalise to other cancer populations. Further studies will be needed to test the stability of the observed predictive effect of metacognitive beliefs on persistent emotional distress across genders and different tumour and prognostic groups. In addition, despite the prospective design, it should be noted that causality can still not be assumed as the influence of unmeasured confounders cannot be ruled out. In order to provide more compelling evidence of a causal role for metacognitive beliefs, further studies are necessary that adopt different approaches to design, such as experimental manipulation.

In summary, the findings of the current study provide promising first evidence that metacognitive beliefs can help to predict anxiety, depression and trauma 1 year after diagnosis of breast and prostate cancer. Furthermore they support the hypothesis that metacognitive beliefs add to the variance explained in persistent anxiety and depression over and above that explained by negative content of thoughts about cancer. Consequently, therapeutic approaches targeting metacognitive beliefs and processes, rather than the content of negative thoughts about cancer, may prove beneficial for preventing persistent emotional distress in these populations.
